# Tunisian Parental Perspectives on Smartphone Use: Assessing Its Impact on Children

**DOI:** 10.1192/j.eurpsy.2025.834

**Published:** 2025-08-26

**Authors:** S. Hamzaoui, K. Mahfoudh, D. Mezri, U. Ouali, A. Aissa, R. Jomli

**Affiliations:** 1 Razi Hospital, Manouba, Tunisia

## Abstract

**Introduction:**

The rapid increase in smartphone usage among minors raises significant concerns about problematic smartphone use in children and adolescents. As the first generations immersed in high-tech media, today’s youth may be more vulnerable to its negative effects compared to older age groups. Understanding these impacts is essential as smartphones become integral to daily life.

**Objectives:**

This study seeks to assess the prevalence of problematic smartphone usage among a group of children, explore its impact on social, emotional, and academic outcomes, identify associated risk factors, and above all evaluate parental awareness and intervention strategies.

**Methods:**

An online questionnaire was administered to parents of children aged from 1 to 18 years. It included sociodemographic information, the Smartphone Addiction Proneness Scale (SAPS) and additional questions designed to explore various aspects of smartphone use.

**Results:**

In total, 100 parents participated in the study. The preliminary results revealed that 60% of children started using smartphones before the age of 6 years and displayed signs of problematic smartphone use, with a notable negative correlation between high usage and academic performance. Additionally, half of parents expressed concerns about their children’s social skills, reporting that excessive smartphone use often diminished social interactions, communication and attention. Withdrawal symptoms were common, with 30% of parents indicating that their children experienced anxiety or restlessness when separated from their devices. Finally, 86% of parents expressed interest in receiving expert advice on healthy and balanced smartphone use for their children.

**Conclusions:**

The results indicate a troubling trend of smartphone addiction among children.This highlights the urgent need for greater parental awareness and active strategies to manage smartphone use. Future research should focus on developing and evaluating intervention programs aimed at fostering healthier technology habits in children.

**Disclosure of Interest:**

None Declared

